# Supplementary material for: Identification and Characterization of a New Platinum-Induced TP53 Mutation in MDAH Ovarian Cancer Cells
Source: Cells. 2019 Dec 21;9(1):36. doi: 10.3390/cells9010036 (PMC7016977; doi:10.3390/cells9010036)
Supplement: Supplementary file 1 [file cells-09-00036-s001.pdf]

Supplementary Information

Identification and Characterization of a New Platinum-Induced TP53 Mutation in MDAH Ovarian Cancer Cells

Ilaria Lorenzon, Ilenia Pellarin, Ilenia Pellizzari, Sara D'Andrea, Barbara Belletti, Maura Sonego [†], Gustavo Baldassarre*[,†], and Monica Schiappacassi *[,†]

Division of Molecular Oncology, Centro di Riferimento Oncologico di Aviano (CRO), IRCCS, National Cancer Institute, Via Franco Gallini, 233081 Aviano, Italy; ilaria.lorenzon@cro.it (I.L.); ilenia.pellarin@cro.it (I.P.); ilenia.pellizzari@gmail.com (I.P.); sdandrea@cro.it (S.D.); bbelletti@cro.it (B.B.); msonego@cro.it (M.S.)

* Correspondence: gbaldassarre@cro.it (G.B.); mschiappacassi@cro.it (M.S.); Tel.: +39-0434-659-759 (ext. 661); Fax: +39-0434-659-428

† These authors contributed equally to this work as senior authors.

Figure S1 related to Figure 1

a

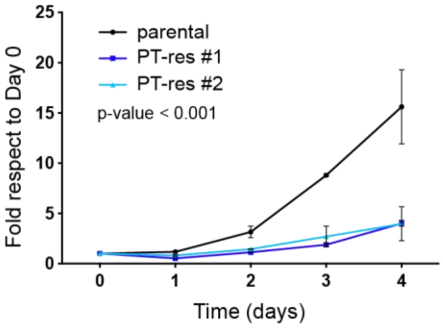

b

% Trypan blue positive cells

cell line	Day 1	Day 2	Day 3	Day 4
parental	0	1.8	2.2	1.3
PT-res #1	6	3.4	4.7	4.2
PT-res #2	0	1.8	6.2	4.7

c

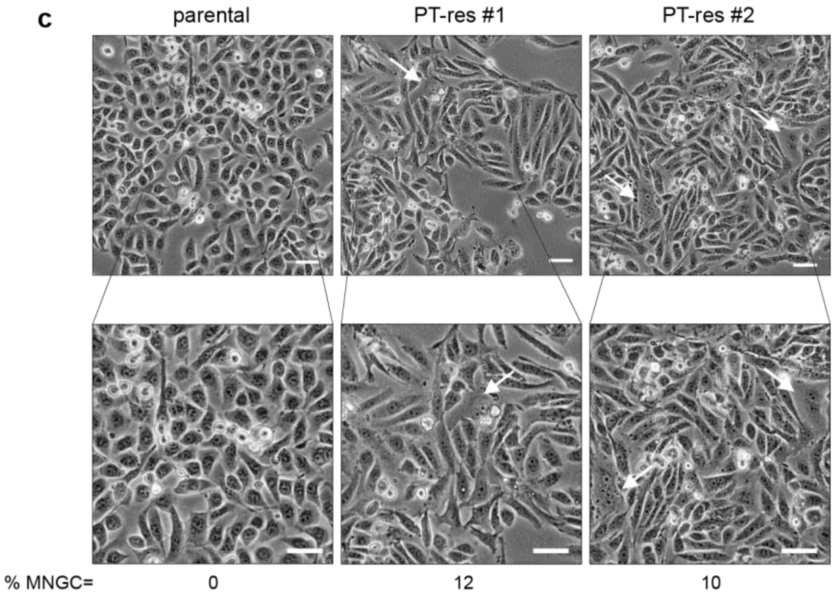

d

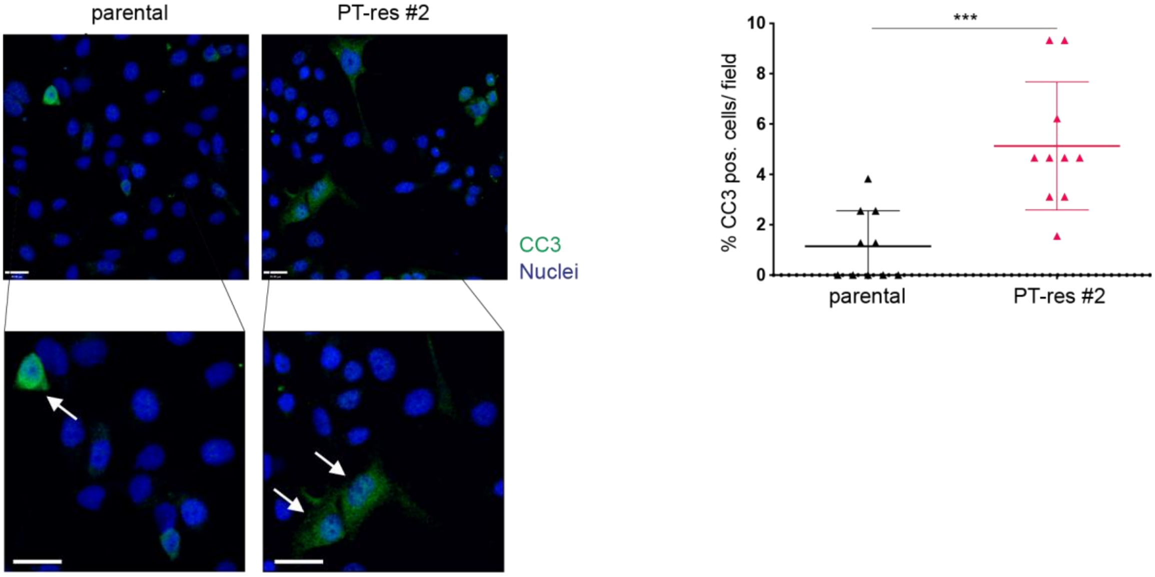

Figure S1.

a) Growth curves of MDAH-2774 parental and PT-res pools. Data are expressed as fold increase respect to day 0. Global statistical significance was determined by two-way ANOVA test.

b) Table reporting the percentage of Trypan blue positive (dead) cells at each time-point of the growth curve (a).

c) Representative phase-contrast photomicrographs of MDAH parental and PT-res pools in exponential growth phase (20X, original magnification). Scale bar = 25 μm. Bottom panels showed a zoomed area for each condition (2× zoom) in which white arrows point to possible MultiNucleated Giant Cells (MNGC) here defined as cells with a cell area 1.5 fold bigger than the mean cell area of parental MDAH cells, as calculated using the ImageJ software. The percentage of MNGC in each cell line is reported under the respective images (at least 50 cells were analyzed for each cell line).

d) Representative images of Cleaved Caspase 3 (CC3) (green) in MDAH parental and PT-res cells cultured in exponentially growing conditions. Nuclei are in blue. Scale bar = 33 μm. Bottom panels showed a zoomed area for each condition (2× zoom) in which white arrows indicate CC3 positive cells. On the right the graph reports the number (mean ± SD) of CC3 positive cells/field is reported. Statistical significance was determined by unpaired Student's t-test (*$p < 0.05$ and **$p < 0.01$ and ***$p < 0.001$).

Figure S2 related to Figure 2

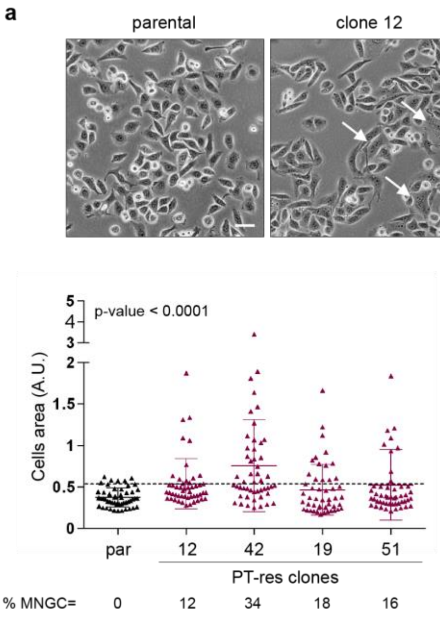

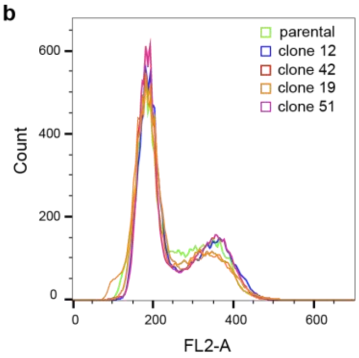

	% cells		
cell line	G1	S	G2/M
parental	42	43	15
clone 12	55	24	22
clone 42	59	23	18
clone 19	56	27	17
clone 51	54	24	22

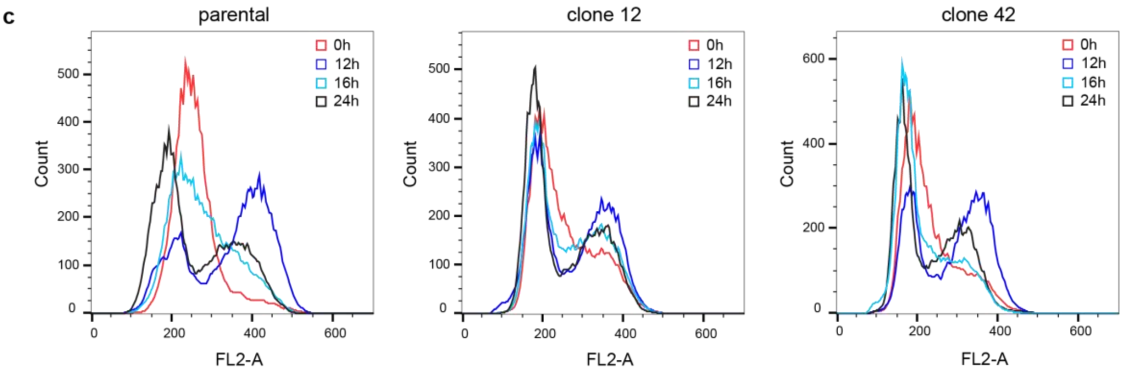

parental

	% cells		
cell line	G1	S	G2/M
0h	0	95	5
12h	16	51	33
16h	10	70	20
24h	39	48	13

clone 12

	% cells		
cell line	G1	S	G2/M
0h	44	55	6
12h	30	23	47
16h	37	43	20
24h	47	26	27

clone 42

	% cells		
cell line	G1	S	G2/M
0h	36	47	17
12h	26	24	50
16h	43	39	18
24h	42	32	26

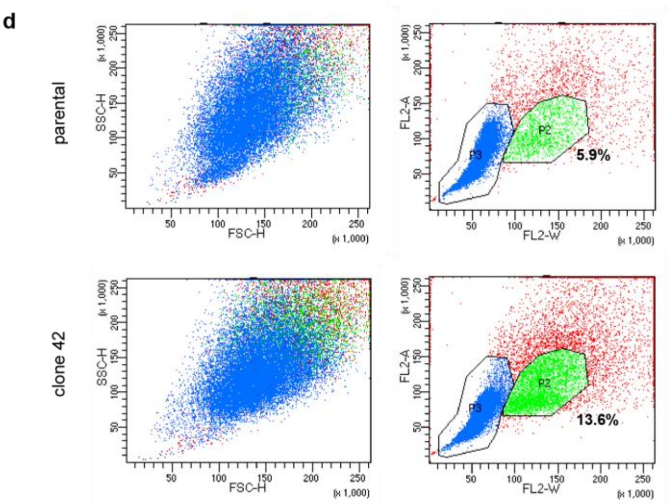

Figure S2.

a) Representative phase-contrast photomicrographs of MDAH parental and PT-res clones in exponential growth phase (20X, original magnification). Scale bar = 25 μm. The graph reports the cell area (mean ± SD) expressed as arbitrary units (A.U.). A threshold has been established (dash line) to determine the percentage of MNGCs cells here defined as cells with a cell area 1.5 fold bigger than the mean cell area of parental MDAH cells. The quantification of MNGCs is reported under the graph as % of analyzed cells. Statistical significance was determined by one-way ANOVA test, global $p < 0.0001$.

b) FACS analyses of DNA content of MDAH parental and PT-res clones in exponentially growing conditions. Live cycling cells were gated using FL2-Width and FL2-Area (see also figure S2d.) The histogram overlay shows the cell cycle distribution of each cell line, as indicated by the colour code. The right table reports the cells percentage in G1, S or G2/M phases in a typical experiment.

c) FACS analyses of DNA content of MDAH parental and PT-res clones (#12 and #42) synchronized by double thymidine block and then released in thymidine free medium for the indicated hours. Cells were gated as in (b). The histogram overlay shows the cell cycle distribution of each cell line at the different time points, as indicated by the colour code. The bottom tables report the cells percentage in G1, S or G2/M phases.

d) FACS analyses of MDAH parental and PT-res clone 42 cells evaluating the size and the cell cycle distribution of analysed living cells. The left panels show cell size as determined by forward and side scatter fluorescence. The right panels show the DNA content distribution evaluated by the analyses of PI fluorescence read as FL2-Width and FL2-Area. In blue (P3) is highlighted the cycling population analysed in the histograms reported in c. In green (P2) is highlighted the giant cells population which also shows a DNA content >2n. The percentage of P2 cells is reported in plots. In red are marked dead cells or cells that cannot be properly classified.

In all analyses Propidium iodide (PI) stained cells were studied using the BD FACScan™ or BD FACSCalibur™ flow cytometers.

Figure S3 related to Figure 2

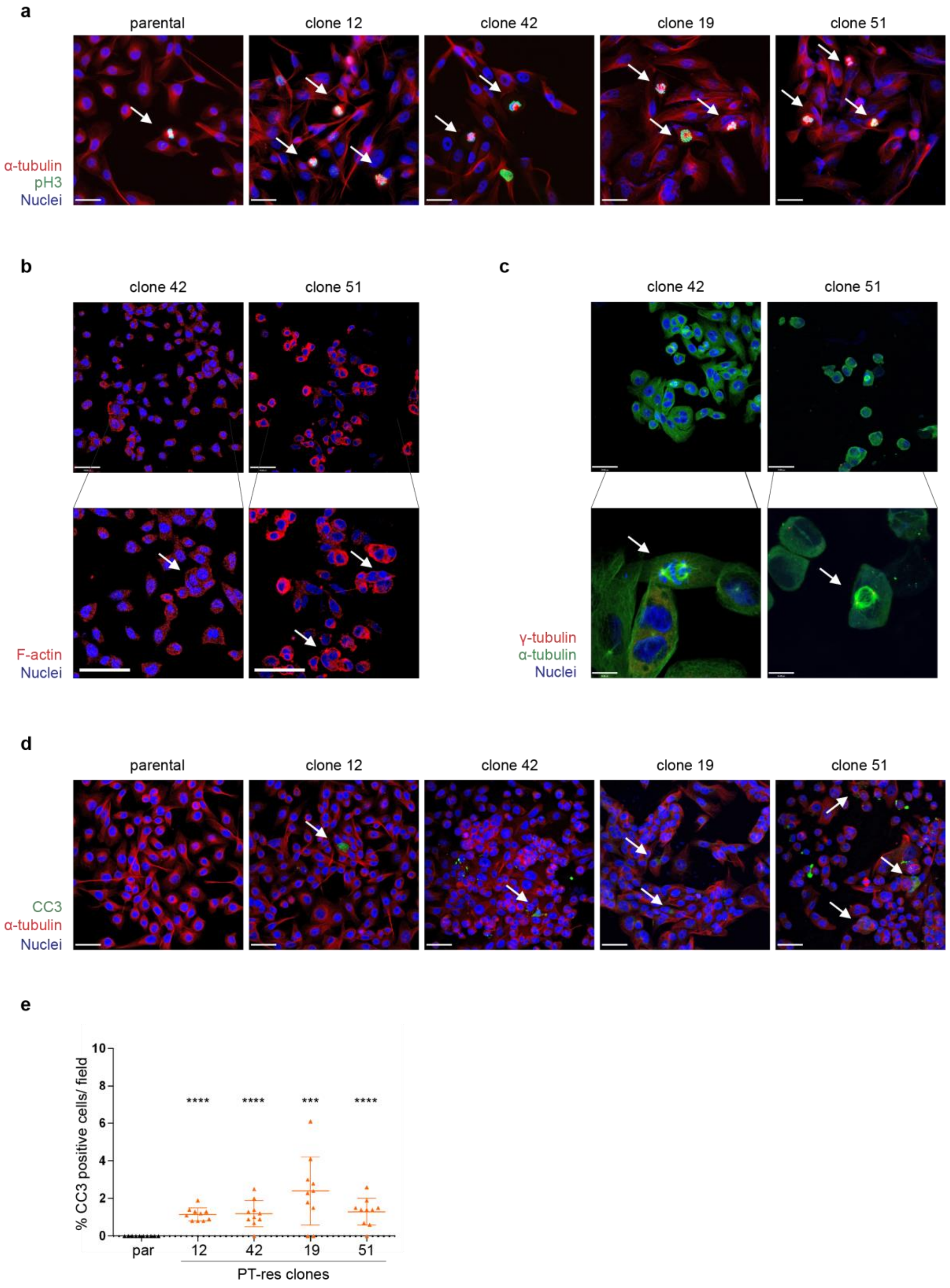

Figure S3.

a) Representative images of MDAH parental and PT-res single clones, immunostained for phospho Histone 3 (pH3, green), α-tubulin (red) and TO-PRO-3 (nuclei, blue) are reported. White arrows indicate pH3-positive mitotic cells. Scale bar = 44 μm.

b) Representative images of MDAH parental and PT-res cell clones stained for F-actin with Phalloidin (F-actin, red). Nuclei are pseudo coloured in blue (TO-PRO-3). Bottom panels show the indicated zoomed area for each condition (2× zoom) in which white arrows indicate multinucleated cells. Scale bar = 44 μm.

c) Representative images of parental and PT-res cell clones, immunostained for α-tubulin (green), γ-tubulin (red) and TO-PRO-3 (nuclei, blue). Scale bar = 44 μm. Bottom panels show the indicated zoomed area for each condition (2× zoom) in which white arrows indicate mitotic cells. Scale bar = 11 μm.

d) Representative images of Cleaved Caspase 3 (CC3) (green) and α-tubulin (red) immunofluorescence staining in MDAH parental and PT-res clones in exponentially growing conditions. Nuclei are pseudo coloured in blue (TO-PRO-3). White arrows indicate CC3 positive cells. Scale bar = 44 μm.

e) Analysis of the immunofluorescence staining showed in (d). The graph reports the number (mean ± SD) of CC3 positive cells/field. Statistical significance was determined by a two-tailed unpaired Student's t-test (*$p < 0.05$ and **$p < 0.01$ and ***$p < 0.001$ and ****$p < 0.0001$).

Figure S4

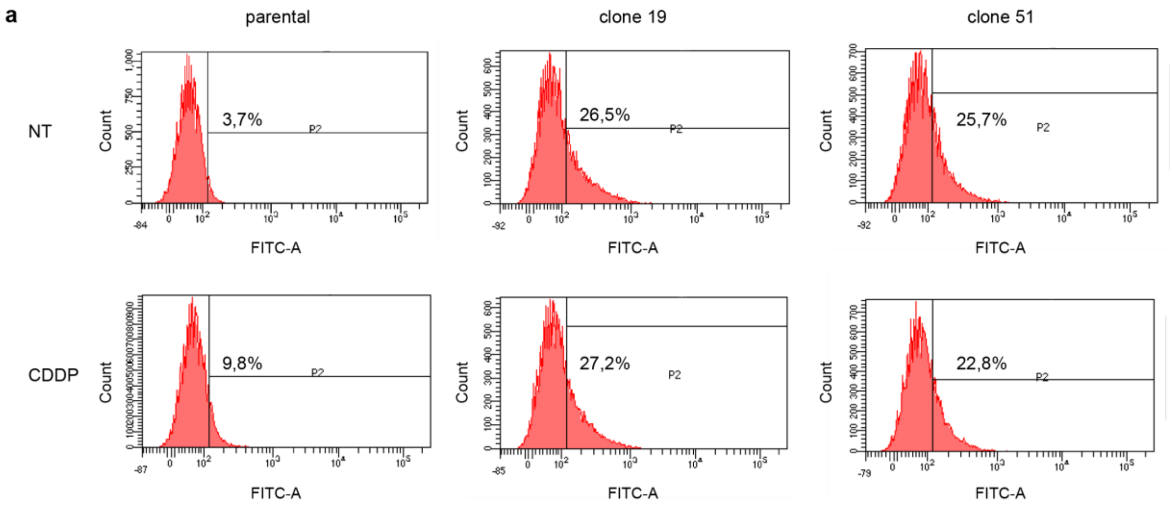

Figure S4. a) Flow cytometry profiles of 2',7'-dichlorodihydrofluorescein diacetate (H_2DCFDA) fluorescence (FITC-A) used as an indicator for reactive oxygen species (ROS) generated in MDAH parental and PT-res clones, treated or not with cisplatin for 24 hours. The emission wavelength (529 nm) was detected and recorded by BD FACSCanto™ II flow cytometer. The percentages of positive cells for oxidized H_2-DCF-DA are indicated in the plots.

Figure S5 related to Figure 3

a

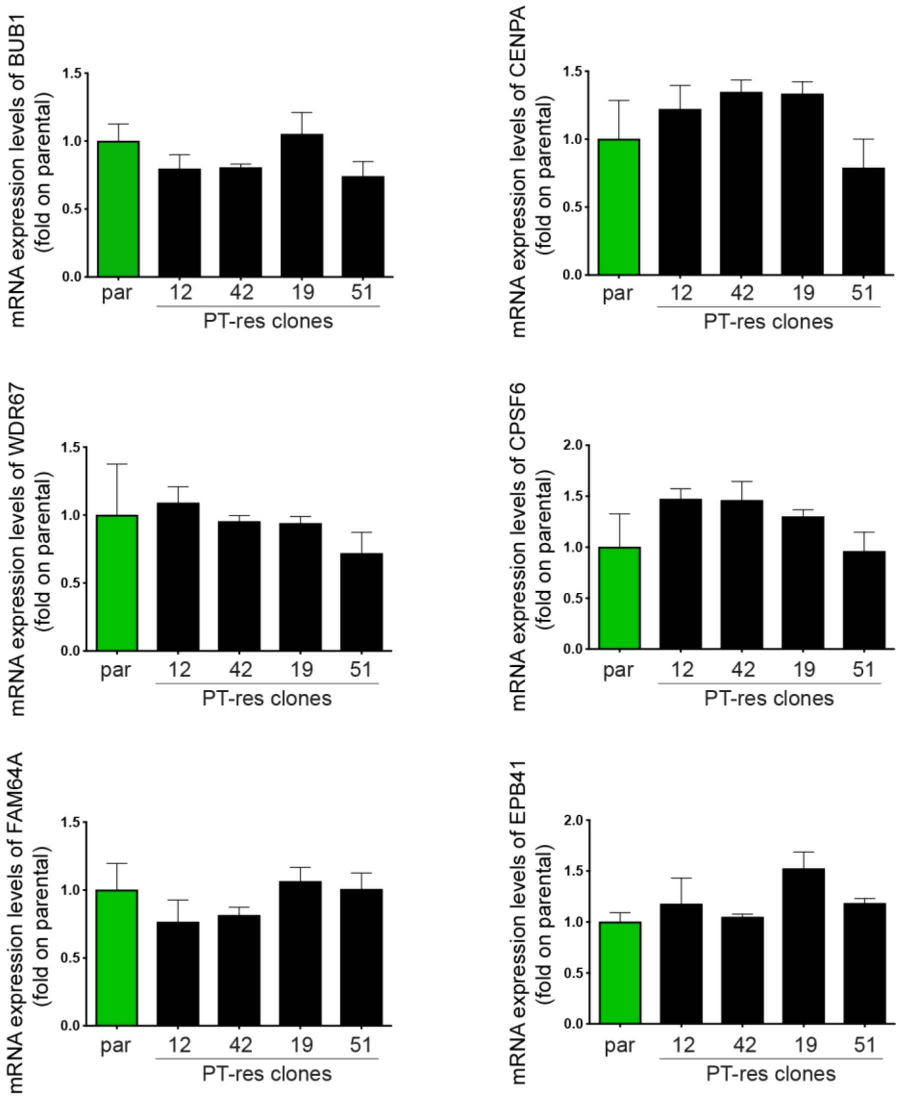

Figure S5. Analyses evaluating the mRNA expression of the indicated p53^{MUT} transcriptional target genes in parental and PT-res single clones. mRNA levels were analyzed in triplicate and normalized using GAPDH as housekeeping gene. Data are expressed as fold of mRNA expression in PT-res cells respect to parental one and represent the mean (± SD) of at least of three independent experiments.

Figure S6

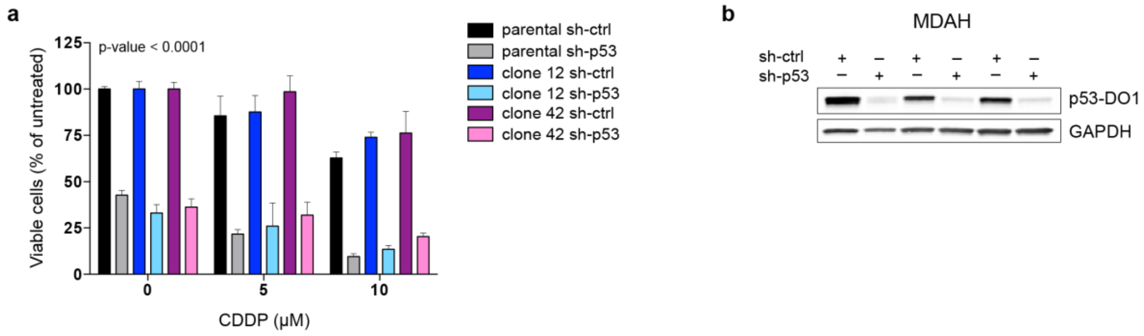

Figure S6.

a) Dose-response curves of MDAH parental and PT-res single cell clones, transduced with sh-ctrl or sh-p53 and treated with increasing doses of cisplatin (CDDP) for 16 hours and released for 24 hours. Data are expressed as percentage of viable cells respect to the sh-ctrl untreated parental cells and represent the mean (±SD) of 3 biological replicates. Statistical significance was determined by two-way ANOVA test; a multiple comparison analysis was done to determine significant differences among groups.

b) Western Blot analysis evaluating p53 expression in parental and PT-res clones, transduced with sh-ctrl or sh-p53 used in (a). GAPDH was used as loading control.

Supplementary Material

List of primers used for Real-time PCR and DNA Sanger Sequence

Primers for Real-time PCR

Name	Sequence (5'-3')
BUB1 f	ATTCAAGCCACAGAGTGGAGCAG
BUB1 r	AGAACTTGTGTTGGCAACCTTATGTG
C21ORF45 f	GCGACTCGCTGAGCTGGGTG
C21ORF45 r	CCCCGCGCAGCACAAAGTCT
CCNE2 f	TGAGCCGAGCGGTAGCTGGT
CCNE2 r	GGGCTGGGGCTGCTGCTTAG
CENPA f	CTTCCTCCCATCAACACAGTCG
CENPA r	TGCTTCTGCTGCCTCTTGTAGG
CPSF6 f	AGGGGCTGTTCCTGGTGGGG
CPSF6 r	GGCCCAGCTAGAGGAGGAGGC
DEPDC1 f	TGGGTATTATCTGCCATGAAGTGCCT
DEPDC1 r	AGGTTGCAGCAAGCCCAAAATGT
EPB41L4B f	CGACGGGACCGAAGTGAGCG
EPB41L4B r	CAGTGCGCAACCTGGGCAGA
FAM64A f	CTCCAGGCTGCAGCTCGCTC
FAM64A r	CAGCCGGGTGCTCTTCTGGC
NCAPH f	GAGGAGCCTGCCCCCTGTCA
NCAPH r	TGGGCCTCCTGCTGCTGACT
WDR67 f	AGGCAACAAGGAGAGCGGCA
WDR67 r	AGCAGTCGCCTGTGCCATCA

Primers for PCR and Sanger sequencing

Name	Sequence (5'-3')
p53 exon5 f	GCCGTCTTCCAGTTGCTTTA
p53 exon5 r	ACACGCAAATTTCCTTCCAC
p53 exon8 f	GGGAGTAGATGGAGCCTGGT
p53 exon8 r	GTTGGGCAGTGCTAGGAAAG
